# Phylogenetic Analysis of Bolivian Bat Trypanosomes of the Subgenus *Schizotrypanum* Based on Cytochrome *b* Sequence and Minicircle Analyses

**DOI:** 10.1371/journal.pone.0036578

**Published:** 2012-05-09

**Authors:** Lineth García, Sylvia Ortiz, Gonzalo Osorio, Mary Cruz Torrico, Faustino Torrico, Aldo Solari

**Affiliations:** 1 Instituto de Investigaciones Biomédicas IIBISMED, Facultad de Medicina, Universidad Mayor de San Simón, Cochabamba, Bolivia; 2 Programa de Biología Celular y Molecular, ICBM, Facultad de Medicina, Universidad de Chile, Santiago, Chile; 3 Programa de Microbiología, ICBM, Facultad de Medicina, Universidad de Chile, Santiago, Chile; Mahidol Oxford Tropical Medicine Research Unit, Thailand

## Abstract

The aim of this study was to establish the phylogenetic relationships of trypanosomes present in blood samples of Bolivian *Carollia* bats. Eighteen cloned stocks were isolated from 115 bats belonging to *Carollia perspicillata* (Phyllostomidae) from three Amazonian areas of the Chapare Province of Bolivia and studied by xenodiagnosis using the vectors *Rhodnius robustus* and *Triatoma infestans* (*Trypanosoma cruzi marenkellei*) or haemoculture (*Trypanosoma dionisii*). The PCR DNA amplified was analyzed by nucleotide sequences of maxicircles encoding cytochrome b and by means of the molecular size of hyper variable regions of minicircles. Ten samples were classified as *Trypanosoma cruzi marinkellei* and 8 samples as *Trypanosoma dionisii*. The two species have a different molecular size profile with respect to the amplified regions of minicircles and also with respect to *Trypanosoma cruzi* and *Trypanosoma rangeli* used for comparative purpose. We conclude the presence of two species of bat trypanosomes in these samples, which can clearly be identified by the methods used in this study. The presence of these trypanosomes in Amazonian bats is discussed.

## Introduction

Short-tailed bats of the genus *Carollia* are widely distributed in the New World tropics. Also, there are detailed altitudinal records from the Peruvian Andes and samples of the three South American species found on both sides of the Andes are available, allowing testing of models of diversification across the Andes. Given the ability to fly, it would be expected dispersal might be expected to play a stronger role than vicariance in shaping bat phylogeographical patterns of variation [1]. Although *Artibeus, Carollia*, and *Glossophaga* generally feed on plant sources, it is clear that they also frequently consume significant quantities of insects [2]. Bats play a crucial role in tropical ecosystems by dispersing seeds, pollinating flowers, and controlling insect populations. *C. perspicillata* may be considered as understorey specialists (from 0–2.5 m high). The short-tailed fruit-eating bats, *C. perspicillata* and *C. brevicauda*, feed primarily on understorey plants such as *Piper, Solanum* and *Vismia* [3]. Roosting habits of these bats are caves, abandoned mine and rail tunnels, active road tunnel, hollow trees, drain pipes and culverts, unused/abandoned buildings or rooms, attics, basements, under bridges, unused cisterns, darkened recesses in rock formations or stream banks. Although there is limited field and experimental evidence, haematophagous arthropods can act as vectors of trypanosomes among bats [2]. Trypanosomes (genus *Trypanosoma*) are widespread blood parasites of vertebrates, usually transmitted by arthropod or leech vectors. Most trypanosome-infected bats are insectivorous and infection could also occur through the ingestion of infected arthropods. Bats are long-lived species and infections persist for years, with trypanosomes localising in skeletal, cardiac and stomach muscle cells [4], [5]. Variable prevalence of trypanosomes in bats has been reported in surveys conducted throughout the world. In South American bats, prevalence varied widely. Colombian bats had a prevalence of approximately 9.0% infected with *Schizotrypanum* spp. [6], [7]. Surveys performed in the Amazonia of Brazil; detected trypanosomes prevalence of 2.4–4.6%, by means of blood smears [8], [9]. The strong association between *Chiroptera* order and all *Schizotrypanum* spp. suggests a long shared evolutionary history. Trypanosomatids parasitize many vertebrate and invertebrate phyla. Several trypanosome species are agents of disease in humans and/or livestock particularly in the tropics. For example, *Trypanosoma brucei* causes human African trypanosomiasis or sleeping sickness, while *Trypanosoma cruzi* causes Chagas disease in South and Central America. There is also strengthened support for two deep clades, one comprising a wide selection of mammalian trypanosomes and a tsetse fly-transmitted reptilian trypanosome, and the other combining two bird trypanosome subclades. Most clades are associated with a type of vertebrate or invertebrate host, or both, indicating that ‘host fitting’ has been the principal mechanism for evolution of trypanosomes [10]. The type species of the subgenus *Schizotrypanum* is *T. cruzi*, which infects man and a wide variety of mammalian hosts. Six different *T. cruzi* lineages have been described, named TcI-TcVI [11]. In the southern cone of South America, isolates from humans and vectors of domestic and peridomestic transmission cycles are predominantly of lineages TcII,Tc V and Tc VI. Tc I and Tc bat have been reported in the sylvatic cycle throughout Latin America (Tc I present in bat genus such as *Thyroptera, Carollia* and Tc bat in *Myotis, Noctilio*). Tc I predominantly infects humans in endemic areas northwest of the Amazon basin [12]. In contrast, all other species traditionally classified as *Schizotrypanum* are restricted to bats. *Trypanosoma cruzi marinkellei* is indigenous to South and Central America, and restricted to bats [13]. *T. c. marinkellei* is, apparently, only transmitted by triatomines of the genus *Cavernicola*, which is found associated with bat colonies in caverns, hollow trees and palms [6], [5]. Also strains of *Trypanosoma vespertilionis* and *Trypanosoma dionisii* from European bats have been distinguished from other *Schizotrypanum* species [13], [14]. *T. dionisii*, *T.c. marinkellei* and *T. cruzi*, belonging to the subgenus *Schizotrypanum*, can invade mammalian cells. These *Trypanosoma* species display distinct surface profiles but invade host cells through a common mechanism involving lysosome mobilization to the site of parasite entry [15]. Anti -*T. dionisii* monoclonal antibodies were tested against various strains of *T. dionisii*, *T. vespertilionis*, *T. cruzi* and *T. c. marinkellei*. The cross reactions between *T. dionisii* and *T. cruzi* demonstrate a strong correlation between *T. dionisii* and TcII-TcVI. Similarly TcI and *T. c. marinkellei* show very similar antigenic pattern [16]. The subgenus *Schizotrypanum* includes several trypanosome species that are difficult to discriminate by morphological examination [17]. Molecular phylogenetic data based on the SSU rRNA indicated that the broad host-range trypanosome *Trypanosoma rangeli* and the rat trypanosome *Trypanosoma cornohini* should also be reclassified in the subgenus *Schizotrypanum* [18]. *T. rangeli* are kinetoplastid protozoa which have been largely recognized and defined in several Latin American countries in relation to *T. cruzi*, because the two trypanosome species are frequently found in mixed infections in triatominae vectors, humans and a variety of wild and domestic mammals [19].

Trypanosomes are protozoa belonging to the *Kinetoplastida* order. The characteristic of this order is a highly unusual, concatenated mitochondrial DNA structure, the kinetoplast DNA (kDNA). Two types of DNA molecules are present, the maxicircles and minicircles. The maxicircles are 22,000 to 33,000 bp in size; they encode mitochondrial proteins. Along with other mitochondrial genes cytochrome b (cytB) are present in 10 to 20 identical copies. The cytB genes are transcribed but they suffer a posttranscriptional modification at the 5′end called editing, in which the mature messenger RNA changes its sequence by multiple insertions and deletions of uridines [20]. In contrast, minicircles are highly heterogeneous in nucleotide sequence; however, the size of minicircles is virtually conserved in *T. cruzi* populations [21]. Restriction endonuclease and sequence analyses showed that a *T. cruzi* minicircle is composed of 4,3,2 or 1 conserved regions of approximately 100 to 150 bp that contain 3 hyper conserved sequence blocks used as universal probes, which are flanked by variable regions with sequences that diverge almost completely as determined in *T. cruzi* and *T. rangeli* [22]. No information is available about trypanosomes minicircles size circulating in bats. With the goal to establish the phylogenetic relationships of trypanosomes present in blood samples of Bolivian *Carollia* bats, we determined the nucleotide sequence of a portion of the cytB gene and characterized the size of the minicircle variable region in trypanosome stocks isolated from Amazonian bats of Bolivia. We include in this work *T. cruzi* and *Schizotrypanum* stocks available information of the cytB in GenBank from Brazilian bats for comparative purposes.

## Methods

### Origin of the Stocks and Ethics Statement

Bats were captured and manipulated using nets and procedures permitted by the Viceministerio de Medio Ambiente, Biodiversidad, Cambios Climáticos y Gestión y Desarrollo Forestal of Bolivia.

Peripheral blood samples were taken from all bats through xenodiagnosis to further culture in NNN agar medium for *T.c. marenkellei* isolation and haemoculture for *T.dionisii*. All bats were analyzed by xenodiagnosis using five nymphs each of the species *R. robustus* and *T. infestans*. All positive isolates were finally cloned [23] and harvested by centrifugation in the log phase. DNA purification was performed using the High Pure DNA preparation kit from ROCHE according to the manufacturer instructions.

### PCR Amplification and Analysis of Cytochrome b Sequences

PCR amplification and sequencing of the partial sequences (≈ 516 bp) of cytB from eighteen bat isolates was performed as described previously. The primers used for amplification of the 5′ half of cytB were: p18 (5′-GACAGGATTGAGAAGCGAGAGAG-3′) and p20 (5′-CAAACCTATCACAAAAAGCATCTG-3′). Reaction conditions were the same as described before. [24]. Thirty-five cycles (94°C, 1 min; 50°C, 30 s; 72°C, 90 s) followed by a final elongation step (5 min, 72°C) were performed. Sequence determination of PCR products was carried out with the Dye Terminator Cycle Sequencing Ready Reaction kit (Perkin-Elmer) on an ABI-373 Automated DNA Sequencer. Sequences of bat trypanosomes derived from this study were aligned with sequences determined in previous studies of bat *T. cruzi* stocks, other trypanosomes isolated from bats and *T. rangeli* available in GenBank [24], [25]. Sequences obtained from this work have accession numbers JN651278 to JN651295. Reference sequences used for tree construction are the following: FJ900248, FJ002262, FJ900247, AJ130927, AJ130932, AJ130933, EU856368, AJ439725, AJ439721, FJ555642, FJ555651, FJ002261, FJ002258, AJ130938, FJ549392, FJ555639, FJ900255, FJ002263 and FJ900249. Sequences Tcm B3 and Tcm B34 were provided directly by Dr. S. Brisse [24]. The evolutionary history was inferred by using the Maximum Likelihood method based on the Tamura-Nei model. A discrete Gamma distribution was used to model evolutionary rate differences among sites (5 categories). All positions containing gaps and missing data were eliminated. There were a total of 382 positions in the final dataset. Alignments were made using ClustalW and manually refined. Phylogenetic analysis was performed using maximum likelihood (ML) method (using the Kimura two-parameter model) listed in the MEGA 5.05 analytical package.

### Minicircle PCR Assay

The amplification reactions were performed in triplicate with oligonucleotides 121 (5′-AAATAATGTACGGGT/GGAGATGCATGA-3′) and 122 (5′- GGTTCGATTGGGGTTGGTGTAATATA-3′), which anneal to the four conserved regions present in trypanosomes minicircles [26]. The DNA samples for PCR were boiled for 15 minutes and 5 µl of supernatant was used as DNA template in 50 µl final volumen [22]. Each experiment included a negative control that contained water instead of DNA and a positive control that contained purified DNA of *T. cruzi*. The PCR products were analyzed by electrophoresis in 2% agarose gels and visualized by staining with ethidium bromide.

## Results

**Figure 1:**
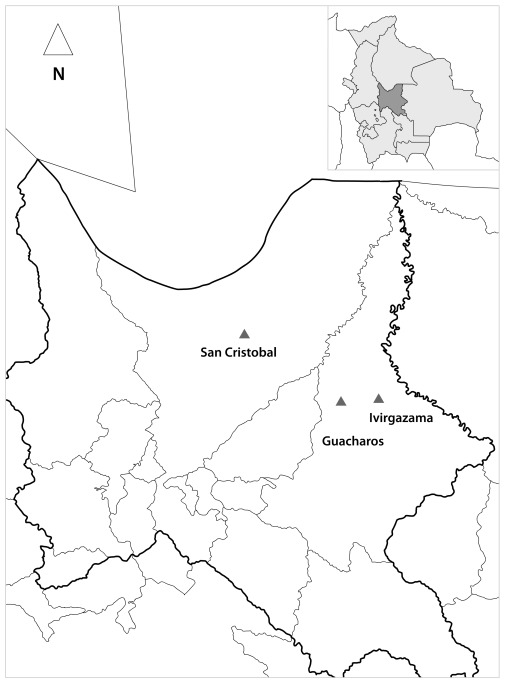
Geographic origin of Trypanosomes isolated from bats in the Amazonian Bioma of Province Chapare, Bolivia.

**Figure 2:**
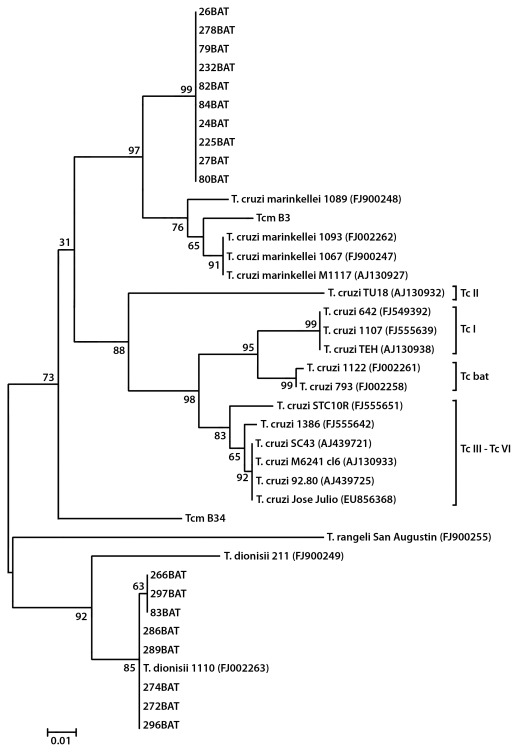
Phylogenetic analysis of sequences of the mitochondrial cytochrome b (cyt B) gene of *Trypanosomatidae* isolated from bats. The evolutionary history was inferred by using the Maximum Likelihood method based on the Tamura-Nei model. Evolutionary analyses were conducted in MEGA5. Numbers at nodes are bootstrap values derived from 1000 replicates. For sequence information see Materials and Methods section.

**Figure 3:**
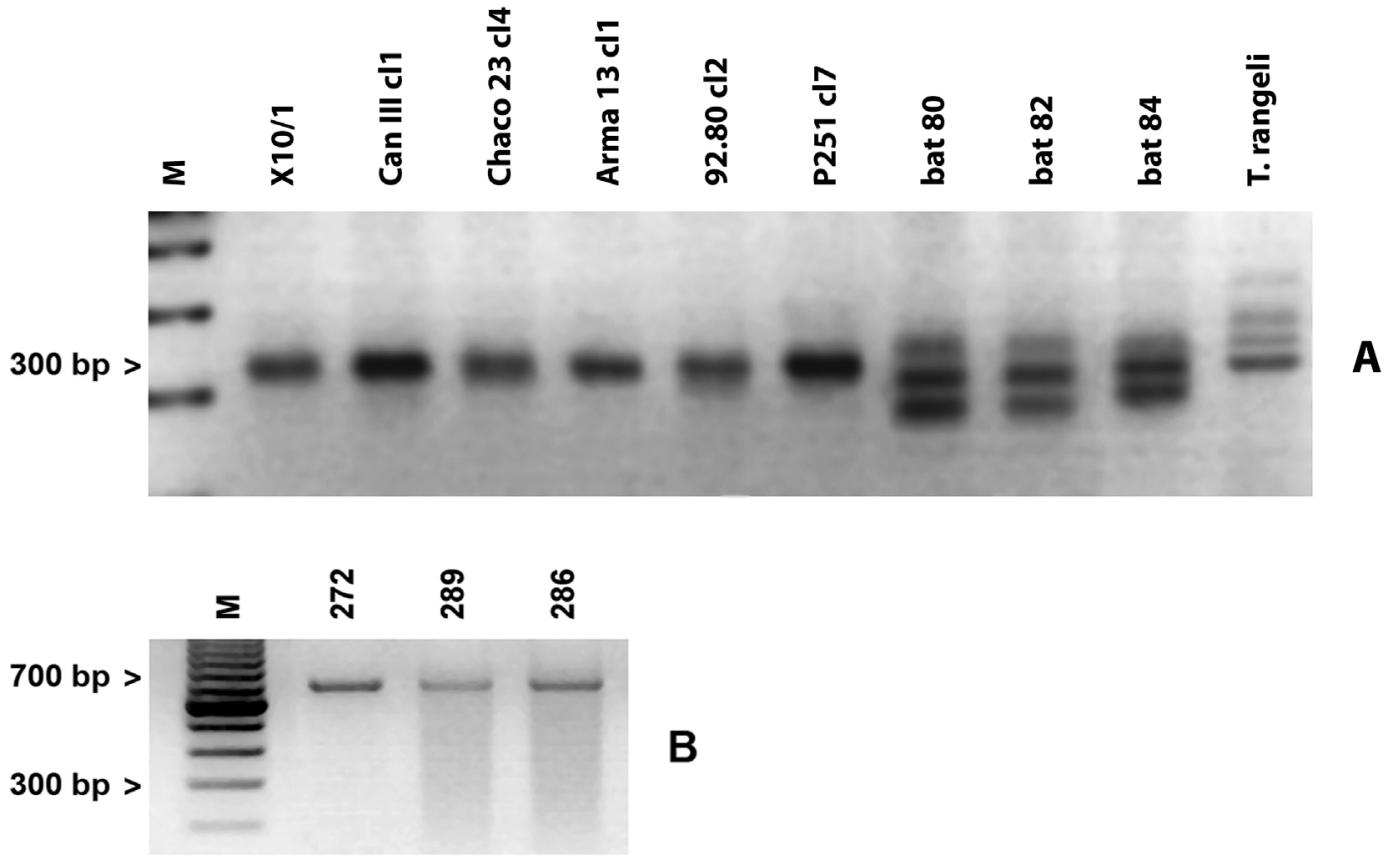
Minicircle PCR amplified analysis with primers 121–122. A. Electrophoresis pattern obtained for 10 Trypanosome stocks: six *T. cruzi* reference stocks DTUs I-VI (lanes 2–7); three *T.c. marinkellei* representative samples from this study (lanes 8–10); one *T. rangeli* reference stock (lane 11). B. Profiles obtained for three *T. dionisii* samples from this study (lanes 2–4); M: molecular weight marker.

A total of 115 bats were caught in three Amazonian areas of Chapare Province, Bolivia [Fig. 1]. All 22 bats from San Cristobal, belongs to *Carollia perspicillata* species. From 24 bats found at Ivirgarzama, 2 were *Desmodus rotundus*, 2 *Glossophaga soricina* and 20 *C. perspicillata*. In Guacharos, 68 bats were found; 6 *Platyrrhinus helleri, 14 Desmodus rotundus* and 48 *C. perspicillata.* All 18 trypanosomes isolated in this study were recovered from bats belonging to *C. perspicillata* the most abundant species in the area (78%). *Tc. marinkellei* was present in bats from San Cristóbal (5), Ivirgarzama (4) and Guacharos (1). *T. dionisii* was isolated only from bats caught in Ivirgarzama (1) and Guacharos (7) [Table 1]. Isolates of *T. dionisii* were obtained only by haemoculture, while T.c. *marinkellei* isolates were obtained by haemoculture, and xenodiagnosis, showing that these species were able to establish infection in triatomines of the genus *Rhodnius,* the endemic triatomine species in the studied Amazonian biodeme. There was no positive xenodiagnosis when *T. infestans* was used. The comparison of sequences from Bolivian bat isolates with those previously studied from Brazil allowed the classification of the isolates in two species, *T. c. marinkellei* and *T. dionisii*; both equally frequent in infected bats. Ten trypanosome isolates (24, 26, 27, 79, 80, 82, 84, 225, 232 and 278) from Bolivian bats grouped closely with the Brazilian stocks of *T. c. marinkellei* 1089 (FJ900248), 1093 (FJ002262) and 1067 (FJ900247), even though they form a separate cluster with a high bootstrap value and all had the same haplotype. Only the reference strain Tcm 34 clustered outside.Other isolates (83, 266, 297, 272, 274, 286, 289 and 296) grouped closely with the *T. dionisii* stock 1110 (FJ 002263) from Brazil and more distantly with the *T. dionisii* stock 211 (FJ 900249) from Brazil [Fig. 2]. This figure also shows phylogenetic relationships between *T. cruzi* clones and *T. cruzi* from bats (Tc bat), including the *T. rangeli* San Agustín stock (FJ 900255). The total DNA of each bat trypanosome isolates and *T. cruzi* clone was used as a template for minicircle PCR amplification with the universal primers 121 and 122 which align in the hyper conserved sequences present in all trypanosomes. The *T. cruzi* clones belonging to all DTUs (Tc I-TcVI) displayed a unique band close to 330 bp, while the PCR of a *T. rangeli* isolate generated bands close to 330 and 380 bp [Fig. 3]. However, isolates 80, 82, 84 belonging to *T. c. marinkellei* and 24, 26, 27, 79, Tcm 1909, Tcm B3 (not shown) displayed a different pattern of amplicons close to 280 and 350 bp. Other bat isolates belonging to *T. dionisii*, on the contrary, displayed a unique band around 600 bp. Results not shown indicate the smaller size of the cultured forms of the Bolivian *T. dionisii* isolates compared to those of *T. c. marinkellei*.

**Table 1:** Sample identification, geographical origin, reservoir and Trypanosome species studied and minicircle PCR assay.

Sample	Geographical origin	Reservoir	Trypanosome species	Minicircle PCR
24	Bolivia, San Cristóbal	*Carollia perspicillata*	*T. cruzi marinkellei*	
26	Bolivia, San Cristóbal	*Carollia perspicillata*	*T. cruzi marinkellei*	
27	Bolivia, San Cristóbal	*Carollia perspicillata*	*T. cruzi marinkellei*	
79	Bolivia, Ivirgarzama	*Carollia perspicillata*	*T. cruzi marinkellei*	
80	Bolivia, Ivirgarzama	*Carollia perspicillata*	*T. cruzi marinkellei*	Fig 3A lane8
82	Bolivia Ivirgarzama	*Carollia perspicillata*	*T. cruzi marinkellei*	Fig 3A lane9
278	Bolivia, Guacharos	*Carollia perspicillata*	*T. cruzi marinkellei*	
225	Bolivia, San Cristóbal	*Carollia perspicillata*	*T. cruzi marinkellei*	
232	Bolivia, San Cristóbal	*Carollia perspicillata*	*T. cruzi marinkellei*	
84	Bolivia, Ivirgarzama	*Carollia perspicillata*	*T. cruzi marinkellei*	Fig 3A lane10
83	Bolivia, Ivirgarzama	*Carollia perspicillata*	*T. dionisii*	
266	Bolivia, Guacharos	*Carollia perspicillata*	*T. dionisii*	
272	Bolivia, Guacharos	*Carollia perspicillata*	*T. dionisii*	Fig 3B lane2
274	Bolivia, Guacharos	*Carollia perspicillata*	*T. dionisii*	
286	Bolivia, Guacharos	*Carollia perspicillata*	*T. dionisii*	Fig 3B lane4
289	Bolivia, Guacharos	*Carollia perspicillata*	*T. dionisii*	Fig 3B lane3
296	Bolivia, Guacharos	*Carollia perspicillata*	*T. dionisii*	
297	Bolivia, Guacharos	*Carollia perspicillata*	*T. dionisii*	
Tcm B3	Brazil, Sao Felipe, Bahia	*Phyllostomus discolor*	*T. cruzi marinkellei*	
TcmM1909	Venezuela, Caracas	*Phyllostomus discolor*	*T. cruzi marinkellei*	
X10/1	Brazil, Belém	*Homo sapiens*	*T. cruzi* I	Fig 3A lane2
*Can III cl1*	*Brazil, Belém*	*Homo sapiens*	*T. cruzi IV*	*Fig 3A* * lane3*
*Chaco 23 cl4*	*Paraguay, Chaco*	*Triatoma infestans*	*T. cruzi II*	*Fig 3A* * lane4*
*Arma 13 cl1*	*Paraguay, Campo Lorro*	*Dasypus novemcinctus*	*T. cruzi III*	*Fig 3A* * lane5*
*92.80 cl2*	*Bolivia, Santa Cruz*	*Homo sapiens*	*T. cruzi V*	*Fig 3A* * lane6*
*P251 cl7*	*Bolivia, Cochabamba*	*Homo sapiens*	*T. cruzi VI*	*Fig 3A* * lane7*
*LDG*	*Colombia, Antioquia*	*Homo sapiens*	*T. T. rangeli*	*Fig 3A* * lane11*

## Discussion

We show in this work that *T. cruzi* is different from *T.c. marinkellei* and *T. dionisii* in the minicircle variable region size. We used the faster-evolving gene cytB to investigate further the genetic distinctness and phylogenetic relationships among *Schizotrypanum* taxa. Phylogenetic relationships among cytB sequences have recently been shown to be congruent with rRNA promoter sequence data [27]. CytB phylogenetic analysis fully supported the high distinctness among *T. rangeli*, *T. dionisii, T. cruzi* and *T. c. marinkellei*. Bolivian trypanosomes from bats, studied here, were grouped as *T. c. marinkellei* or *T. dionisii*, in contrast to Brazil, which also includes the *T. cruzi* lineages TcI, TcII and TcIII (present in bat genus such as *Carollia, Myotis, Noctilio* and *Thyroptera*) [25], and *T. rangeli* as described [28], [29]. All taxa appeared to be roughly equidistant in our analysis, with the exception of *T. cruzi* and *T. c. marinkellei*, which appeared to be more closely related, as described for Brazilian bat trypanosomes.

However the Bolivian *T. c. marinkellei* are genetically distant from the Brazilian ones, indicating that trypanosomes of this species are genetically heterogeneous as described [24]. Most of bats studied here belong to the family *Phyllostomidae*, which exhibit varied alimentary habits, including insectivorous, therefore this represents the probable infection route by feeding of *Cavernicola* triatomines. *A* common ancestry of *T. cruzi* and *T.c.marinkellei* was suggested by 18S rRNA data [18] and phylogenetic analyses demonstrated that Tc bat indeed belongs to *T. cruzi* and not to other closely related bat trypanosomes of the subgenus *Schizotrypanum*, and that although separated by large genetic distances Tcbat is closest to lineage Tc I [12].

In the present study, comprising a survey for bat trypanosomes in an Amazonian biome of Bolivia, the majority of cultures were identified as *T.c. marinkellei* and *T. dionisii* based on cytB gene. The prevalence of *T.c. marinkellei* was 9.0% and 7% of *T. dionisii*. The results strongly supported the suitability of this sequence for analysis of phylogenetic relationships among *Schizotrypanum,* as previously demonstrated for other clades of trypanosomes from mammals [30].

Phylogenetic relationships inferred using ssrRNA, gGAPDH and cytB generated trees with similar topologies and were also congruent with results based on cytB sequences. Three major clades of bat trypanosomes within the subgenus *Schizotrypanum* were strongly supported in all phylogenies regardless of data sets and analytical methods in which the clade containing *T. cruzi* was closer to that containing *T.c. marinkellei* than to *T. dionisii.* No other species of *Schizotrypanum* besides these species before mentioned were isolated from bats in this study, suggesting that other species of this subgenus are rare in this area of Bolivia and/or difficult to cultivate. Closest to the *T. cruzi* clade is *T. rangeli,* another American trypanosome of wild mammals also transmitted by triatomine bugs but rarely found in bats, except in Brazil. Only two cultures of *T. rangeli* from bats have been confirmed using morphological, biological and molecular parameters [29]. Phylogeographical, ecological and biological analyses of isolates classified as *Schizotrypanum* disclosed some patterns of association with bat species, biomes and geographic origin, as well as with their behavior in culture, triatomine bugs and mice. Our results show overlapping geographic areas of the two *Schizotrypanum* species in the Amazonia of Bolivia. *T. c. marinkellei* was found in bats from phyllostomid species (insectivorous, frugivorous) corroborating a strong association with this bat family, as suggested previously [6]. However, bats of this family were also infected by *T. dionisii* as shown in this and other studies [31]. The prevalence of *T. c. marinkellei* may be explained by the abundance of phyllostomid bat, whereas its distribution may be determined by that or its triatomine vector, *Cavernicola pillosa,* which shares caves, holes in trees, and palm leafs with bats.

We have demonstrated the existence in Bolivia of *T. dionsii,* another trypanosome found in neotropical bats. However the scarcity of *T. dionisii* in two areas studied here can be explained by the abundance and distribution of its vectors. The genetic distances of bat trypanosomes provided by this study are better explained by the ability of bats to disperse over large areas, crossing oceans and continents rather than by vicariance events. The reconstruction of the evolutionary histories of parasites has been linked to the comparable histories of their host. Phylogenetic and biogeographic analyses have suggested that Africa is the centre of origin of modern-day bat families, with a Southern Hemisphere origin in the Cretaceous. Two scenarios could account for the dispersal of bats from Africa in the Eocene: northwards dispersal to Eurasia and via Beringia into America or transatlantic dispersal from Africa to America through island hopping or direct flight [32], [33].

The estimates of divergence time based on nuclear and mitochondrial genes suggested that *T. cruzi* may have evolved from bat-restricted trypanosomes 10–20 mya [34]. Limited divergence among *Schizotrypanum* spp. is compatible with recent diversification, and their present day distribution is equally consistent with hypotheses that *T. cruzi* evolved from a bat-restricted trypanosome or vice versa [18], [24].

Comparative analyses performed in this study showed that the morphology of blood and axenic culture forms (data not shown) and minicircle variable region size should be considered as the preliminary parameters to assign trypanosomes to the subgenus *Schizotrypanum.* A broad phylogeographical analysis including to determine the abiotic factors affecting the distribution patterns of flora and fauna, to compare the adaptations of organisms to different environmental conditions, to explain the historical and geographical reasons that determine the distribution of an organism in space and time, to evaluate the biological interactions that affect the distribution pattern of organisms, to recognize pattern of distribution of bat trypanosomes at the regional and global from Africa, Europe and America, is still required to understand the evolutionary history of *Schizotrypanum* and bat trypanosomes in general.
